# Primary cilia suppress Ripk3-mediated necroptosis

**DOI:** 10.1038/s41420-022-01272-2

**Published:** 2022-12-02

**Authors:** Emilia Kieckhöfer, Gisela G. Slaats, Lena K. Ebert, Marie-Christine Albert, Claudia Dafinger, Hamid Kashkar, Thomas Benzing, Bernhard Schermer

**Affiliations:** 1 Department II of Internal Medicine and Center for Molecular Medicine Cologne, University of Cologne, Faculty of Medicine and University Hospital Cologne, Cologne, Germany; 2 CECAD, University of Cologne, Faculty of Medicine and University Hospital Cologne, Cologne, Germany; 3 Department of Nephrology and Hypertension, University Medical Center Utrecht, Utrecht, The Netherlands; 4 Institute for Molecular Immunology, University of Cologne, Faculty of Medicine and University Hospital Cologne, Cologne, Germany

**Keywords:** Necroptosis, Mechanisms of disease, Paediatric kidney disease

## Abstract

Cilia are sensory organelles that project from the surface of almost all cells. Nephronophthisis (NPH) and NPH-related ciliopathies are degenerative genetic diseases caused by mutation of cilia-associated genes. These kidney disorders are characterized by progressive loss of functional tubular epithelial cells which is associated with inflammation, progressive fibrosis, and cyst formation, ultimately leading to end-stage renal disease. However, disease mechanisms remain poorly understood. Here, we show that targeted deletion of cilia in renal epithelial cells enhanced susceptibility to necroptotic cell death under inflammatory conditions. Treatment of non-ciliated cells with tumor necrosis factor (TNF) α and the SMAC mimetic birinapant resulted in Ripk1-dependent cell death, while viability of ciliated cells was almost not affected. Cell death could be enhanced and shifted toward necroptosis by the caspase inhibitor emricasan, which could be blocked by inhibitors of Ripk1 and Ripk3. Moreover, combined treatment of ciliated and non-ciliated cells with TNFα and cycloheximide induced a cell death response that could be partially rescued with emricasan in ciliated cells. In contrast, non-ciliated cells responded with pronounced cell death that was blocked by necroptosis inhibitors. Consistently, combined treatment with interferon-γ and emricasan induced cell death only in non-ciliated cells. Mechanistically, enhanced necroptosis induced by loss of cilia could be explained by induction of Ripk3 and increased abundance of autophagy components, including p62 and LC3 associated with the Ripk1/Ripk3 necrosome. Genetic ablation of cilia in renal tubular epithelial cells in mice resulted in TUNEL positivity and increased expression of Ripk3 in kidney tissue. Moreover, loss of *Nphp1*, the most frequent cause of NPH, further increased susceptibility to necroptosis in non-ciliated epithelial cells, suggesting that necroptosis might contribute to the pathogenesis of the disease. Together, these data provide a link between cilia-related signaling and cell death responses and shed new light on the disease pathogenesis of NPH-related ciliopathies.

## Introduction

Primary cilia are antenna-like sensory organelles that receive signals from the environment, transmit them to the interior of the cell, and thus modulate the response of cells to environmental influences [1–3]. For this purpose, cilia are covered by a highly specialized plasma membrane whose protein composition is precisely regulated [4]. At the base, a cilium is anchored by its basal body, which resembles a modified centriole. The import and export of ciliary proteins are primarily regulated at the transition zone, located between the basal body and the ciliary shaft [5]. Cilia modulate multiple signaling pathways, including Hedgehog, Wnt, Notch, PDGF, and additional GPCR signaling [1]. Dysfunction or loss of the primary cilium inevitably leads to perturbations of these signaling pathways and results in diseases known as ciliopathies [6]. The spectrum of ciliopathies ranges from severe neuronal developmental disorders and retinal or skeletal ciliopathies to endocrinological conditions and hepatic and renal diseases [7]. While most ciliopathies occur as syndromes that affect different organ systems, a significant feature of many ciliopathies is the involvement of the kidneys. Therefore, this large subgroup is also referred to as renal ciliopathies [8].

Among renal ciliopathies, autosomal-dominant polycystic kidney disease (ADPKD) is the most frequent form, with an incidence of 1:1000, typically affecting adults and leading to end-stage kidney failure at the age of 50 to 60 years [9]. In children, nephronophthisis (NPH), an autosomal-recessive renal ciliopathy, is the most frequent genetic cause of renal failure and is responsible for approximately 10% of children requiring dialysis [10]. The renal phenotype of ADPKD and NPHP differs: Kidneys in ADPKD enlarge significantly during the disease and are progressively interspersed with numerous cysts. In contrast, significantly fewer cysts develop in NPH. Here, kidneys are relatively small and characterized by tissue degeneration and interstitial inflammatory fibrosis [11, 12]. Notably, patients with ADPKD or NPH are born without any overt renal phenotype but massively lose renal tubular epithelial cells with disease onset and progression. In ADPKD, apoptosis has been described very early by TUNEL assays [13] and was later found in several animal models of ADPKD (reviewed in ref. [14]). More recently, the role of apoptosis in cyst lumen formation in ADPKD has been suggested [15]. Remarkably, primary cilia appear to be normal or elongated in kidneys of ADPKD mouse models [16–18], while loss of NPHP genes often results in ciliary abnormalities and lower numbers of primary cilia [19–22].

In addition to apoptosis, various pathways of regulated cell death, specifically regulated necrosis (termed necroptosis), have been described [23] that could contribute to tissue defects associated with the loss of primary cilia. In contrast to immunogenically relatively silent apoptotic cell death, necroptosis involves cellular membrane damage, the release of damage-associated molecular patterns (DAMPs), and provokes inflammatory tissue conditions which further enhance inflammatory tissue destruction [24]. The role of necroptosis in the kidney, in particular in acute kidney injury (AKI) induced by ischemia-reperfusion damage or by pharmacological means, is well-established [25]. Necroptosis is typically activated downstream of receptor activation, including death or toll-like receptors, in conditions when caspase-8 is inhibited [26]. Here, the mechanisms of TNFα (tumor necrosis factor α) signaling are best understood [27, 28]: Binding of TNFα to the TNFR1 receptor recruits TRAAD (TNF-receptor-associated death domain), Ripk1 (receptor-interacting serine/threonine kinase 1), Traf2 and Traf5 (TNF-receptor-associated factor 2/5) as well as cIAP1 and cIAP2 (cellular inhibitor of apoptosis1/2). This active complex I initially results in NF-κB (nuclear factor κB) and MAPK (mitogen-activated protein kinases) activation and transcription of pro-survival genes. Dissociation of the receptor from Ripk1 can result in three different types of complex II, each promoting cell death. The apoptotic complex IIa includes TRADD, FADD, and Caspase-8. Complex IIb requires the absence of cIAP1/2 and results in Ripk1- and Casp-8-dependent apoptosis. Upon inhibition of Caspase-8 Ripk1 and Ripk3 (receptor-interacting serine/threonine-protein kinase 1/3) form a complex often called the necrosome (complex IIc) [29]. Subsequently, active Ripk3 phosphorylates its substrate mixed lineage kinase domain-like (Mlkl), which executes cell death. This most likely involves translocation of Mlkl to the plasma membrane and the formation of pores that disrupt membrane integrity [29, 30]. Remarkably, either expression of a kinase-dead mutant Ripk1, loss of Ripk3, or loss of Mlkl protects mice from kidney failure in different scenarios of AKI [31–33]. In addition, synchronized cell death through the ferroptotic pathway has also been demonstrated to contribute to acute damage and to the loss of entire tubular segments [34]. Upon ferroptotic cell death, however, the immunological response might be much milder as compared to necroptosis. Therefore, the kidneys might be able to cope with ferroptotic cell loss more efficiently than with necroptosis [25]. While the different pathways of necroptosis have been extensively studied in AKI, their role in renal ciliopathies and, in particular, their connection with primary cilia remained elusive. Here, we study how primary cilia modulate cell death induced by TNFα in combination with the SMAC mimetic birinapant or cycloheximide (CHX) or by interferon-gamma (IFNγ) under the inhibition of caspase-8, which typically would promote necroptotic death. Remarkably, these conditions do not induce necroptosis in wild-type renal epithelial cells carrying primary cilia, while cells without cilia display an increased susceptibility towards necroptosis and Ripk1-dependent apotosis. Mechanistically, this can be explained by increased expression of Ripk3 and components of the autophagy-lysosomal pathway in cells without cilia. Moreover, the deletion of the major gene involved in NPH in non-ciliated cells further enhanced the susceptibility to necroptosis, supporting the role of necroptotic death in renal ciliopathies.

## Results

### Loss of cilia increases the susceptibility to necroptosis

**Fig. 1:**
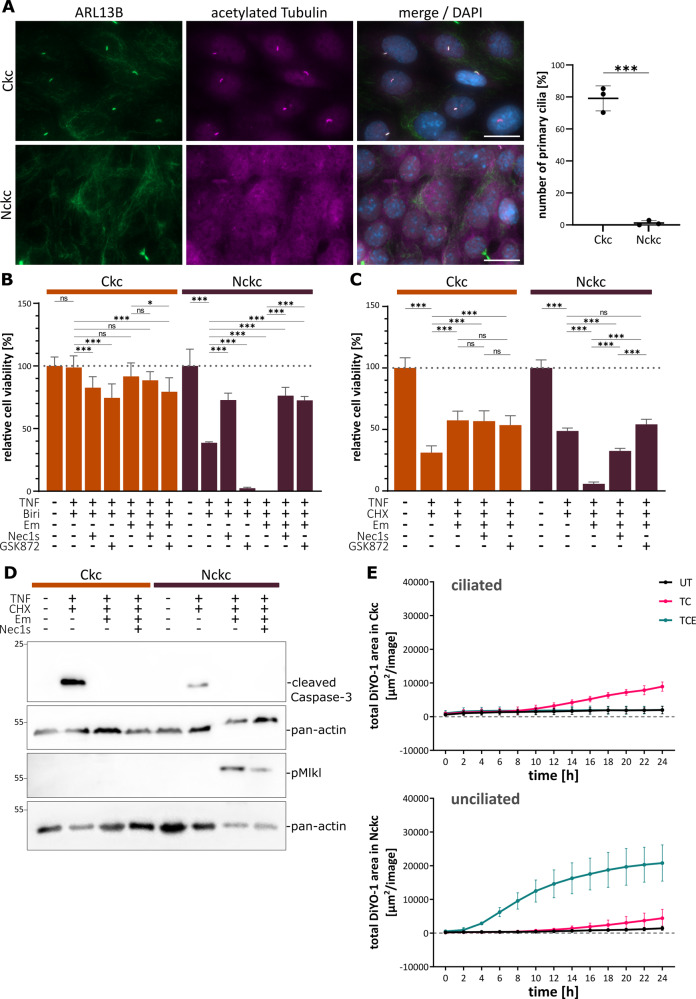
Primary cilia inhibit necroptotic cell death in renal epithelial cells. **A** Immunofluorescence staining of primary cilia in the mIMCD3 subclones Ckc (ciliated kidney cells) and Nckc (non-ciliated kidney cells; ARL13B (magenta), acetylated tubulin (green) and DAPI (blue); scale bar 20 µm). Quantification of cells carrying primary cilia (*n* = 3; total count of 404 cells for Ckc and 613 cells for Nckc). **B** Neutral-red assay in Ckc and Nckc cells after RCD induction with TNFα (TNF, 4 ng/100 µl) and birinapant (biri, 5 µM) for 16 h. Additionally, caspase-8 inhibitor emricasan (Em, 10 µM), Ripk1 inhibitor necrostatin-1s (Nec1s, 40 µM), and Ripk3 inhibitor GSK872 (GSK872, 5 µM) were used for 16 h (*n* = 4). **C** Neutral-red assay in Ckc and Nckc cells after RCD induction with TNFα (TNF, 4 ng/100 µl) and cycloheximide (CHX, 2 µg/100 µl) for 16 h. Additionally, caspase-8 inhibitor emricasan (Em, 10 µM) and necroptosis inhibitor necrostatin-1s (Nec1s, 40 µM) were used for 16 h (*n* = 4). **D** Immunoblot analysis of ciliated and non-ciliated cells using the apoptosis marker cleaved-Caspase-3 (~17 kDa) and the necroptosis marker phospho-Mlkl (~56 kDa). Either pan-actin (~44 kDa) or beta-tubulin (~55 kDa) were used as control (*n* = 3). **E** Live-cell imaging over the period of 24 h after treatment with TNF and CHX (TC), and TNF, CHX, and Em (TCE) or DMSO as control. Cells were stained with the dead cell marker DiYO-1. Images were captured every 2 h (*n* = 3).

To study the role of primary cilia in apoptotic and necroptotic cell death of renal epithelial cells, we used mouse inner medullary collecting duct (mIMCD3) cells, a well-established model in renal and cilia research. Notably, several classical ciliary proteins, including critical components of the intraflagellar transport (IFT) machinery, can affect inflammatory signaling independent of primary cilia [35]. Therefore, instead of targeting proteins involved in IFT to interfere with cilia and ciliogenesis, we generated subclones from the parental wild-type mIMCD3 cell line by FACS and screened those subclones for the presence and absence of primary cilia. We randomly selected two subclones: Ckc (ciliated kidney cells), with about 80% of cells carrying a primary cilium, and Nckc (non-ciliated kidney cells) displaying almost no cilia at all (1%), as demonstrated by cilia staining (Fig. 1A). Induction of Ripk1-dependent cell death with TNFa and the SMAC mimetic birinapant (complex IIb) for 16 hours resulted in Ripk1-dependent cells death and reduced the number of viable cells to 38% in the non-ciliated cells (Fig. 1B). The Ripk1-inhibitor Nec1s partially protected from cell death, while inhibition of Ripk3 with GSK872 further enhanced cells death by inhibiting necroptosis but promoting apoptosis. Combined treatment of non-ciliated-cells with TNFa, birinapant and the caspase-8 inhibitor emricasan [36] resulted in almost no surviving cells. Caspase-8 inhibition is known to unleash necroptotic cell death by involving kinase activity of Ripk1 and Ripk3 [26]. Consistently, this could be almost rescued either by inhibition of Ripk1 or by inhibition of Ripk3, indicating that cell death was caused by nectroptosis. Ciliated cells showed almost no cell death response upon TNFa and birinapant treatment. Here, treatment with Ripk1/3 inhibitors slightly enhanced cell death by promoting apoptosis. Caspase-8 inhibition, which killed almost all non-ciliated cells, had no significant effect on cells with cilia (Fig. 1B). To investigate the role of complex IIa activation, we performed similar assays with induction of cell death by TNFα and CHX (TC) for 16 hours (Fig. 1C). Here, only 24% of cells with primary cilia survived, whereas 44% of non-ciliated cells did not respond to TNFα and CHX, indicating some protection from apoptosis. Remarkably, simultaneous inhibition of caspase activity using emricasan (TCE treatment) positively affected cell survival of ciliated cells (47% viability), while almost all non-ciliated cells underwent cell death (0.3% viability). Consistent with the induction of necroptotic death, the Ripk1-inhibitor Nec1s and the Ripk3 inhibitor GSK872 efficiently reduced TNF-induced cell death only when caspase activity was blocked by emricasan (TCEN treatment) in the non-ciliated cells (Fig. 1C). Immunoblots for cleaved caspase-3 indicated apoptosis occurring primarily in TC-treated ciliated cells, while phospho-Mlkl (pMlkl) as a marker for necroptosis was detected only upon TCE treatment in non-ciliated cells (Fig. 1D). To analyze the temporal dynamics of cell death and the cellular morphology, we performed a live-cell analysis of cells upon treatment with DMSO, TC, or TCE over the period of 24 h. These data confirmed our findings and revealed rapid cell death upon caspase-8 inhibition in the non-ciliated cells already at very early time points (Fig. 1E). Ciliated cells exposed to TC treatment showed membrane blebbing, condensation, and fragmentation of nuclei indicative of apoptosis, while upon TCE treatment, they did neither display nuclear condensation nor formation of apoptotic bodies (Suppl. Fig. 1A). Similarly, dead cells upon TCE treatment of non-ciliated cells did not resemble morphological changes of apoptotic cells. Notably, TNFα-independent induction of cell death via interferon γ (IFNγ) combined with caspase-8 inhibition induced necroptotic death only in cells lacking primary cilia but not in ciliated cells, as shown by additional live-cell assays (Suppl. Fig. 1B).

**Fig. 2:**
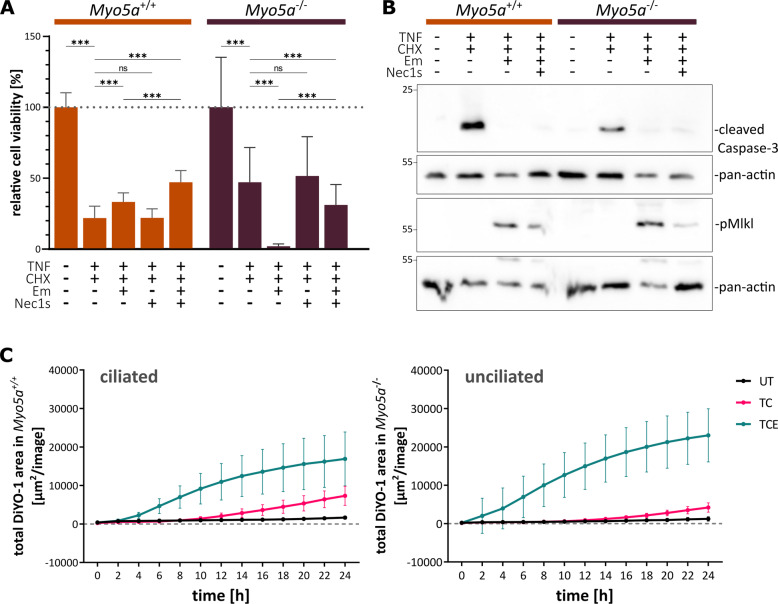
Loss of cilia in Myo5a-deficient cells increases susceptibility to necroptotic death. **A** Neutral-red assay in control and *Myo5a*^−/−^ cells after RCD induction through TNFα (TNF, 4 ng/100 µl) and cycloheximide (CHX, 2 µg/100 µl) for 16 h. Additionally, caspase-8 inhibitor emricasan (Em, 10 µM) and necroptosis inhibitor necrostatin-1s (Nec1s, 40 µM) were used for 16 h (*n* = 4). **B** Immunoblot analysis of ciliated and non-ciliated cells using the apoptosis marker cleaved caspase 3 (~17 kDa) and the necroptosis marker phospho-Mlkl (~56 kDa). As housekeeping control either pan-actin (~30 kDa) or beta-tubulin (~55 kDa) were used (*n* = 3). **C** Live-cell imaging over the period of 24 h after treatment with TNF and CHX (TC), and TNF, CHX, and Em (TCE) or DMSO as control. Cells were stained with the dead cell marker DiYO-1. Images were captured every 2 h (*N* = 8).

For additional confirmation that this switch in the death response resulted from the lack of cilia and to exclude any clonal effects, we used Myosin5a-deficient mIMCD3 cells. Myosin5a (Myo5a) is an actin-based motor and transport protein. Cells deficient in Myo5a are unable to assemble primary cilia [37]. Here, loss of cilia is caused by defective transport of the pre-ciliary vesicle to the mother centriole, the later basal body [38]. Loss of cilia in Myo5a^−/−^ compared to Myo5a^+/+^ control cells was confirmed by immunofluorescence staining using antibodies against Arl13B and acetylated tubulin as ciliary markers (Suppl. Fig. 2A). Cell viability assays with TC, TCE, and TCEN treatments confirmed the findings from the Ckc and Nckc subclones in all aspects: Myo5a^−/−^ cell without cilia were partially protected from apoptosis in response to TC treatment. Induction of necroptosis by TCE treatment led to massive cell death only in Myo5a^−/−^ cells, which again was sensitive to necrostatin-1s (Fig. 2A). Immunoblots confirmed cleavage of caspase-3 primarily in ciliated Myo5a^+/+^ cells which was reduced in Myo5a^−/−^ cells lacking cilia, indicating a lower rate of apoptosis, while the phosphorylation of Mlkl was detectable in both, however slightly increased in non-ciliated cells after caspase inhibition with emricasan (Fig. 2B). Live-cell imaging again revealed the increased occurrence of cell death at a very early time point after TCE treatment (Fig. 2C and Suppl. Fig. 2B). Interestingly, in contrast to the viability assay in Fig. 2A ciliated cells also underwent cell death upon TCE, although to a lower rate as non-ciliated cells. This can be explained by the low cell density required for live-cell imaging, which results in a higher number of proliferating and, therefore, transiently non-ciliated cells as compared to the viability assays. In contrast to the subclones Ckc and Nckc, differences between Myo5a^+/+^ and Myo5a^−/−^ cells, in general, were slightly less pronounced, which might be due to the fact that the number of ciliated cells in the parental Myo5a^+/+^ cells (25%) was much lower (Suppl. Fig. 2A) as compared to the ciliated subclone Ckc used in Fig. 1 (79%; Fig. 1A). Taken together, these data show that loss of cilia results in a shift from apoptotic to necroptotic cell death.

### Altered Ripk3 and Ripk1 in cells lacking primary cilia

**Fig. 3:**
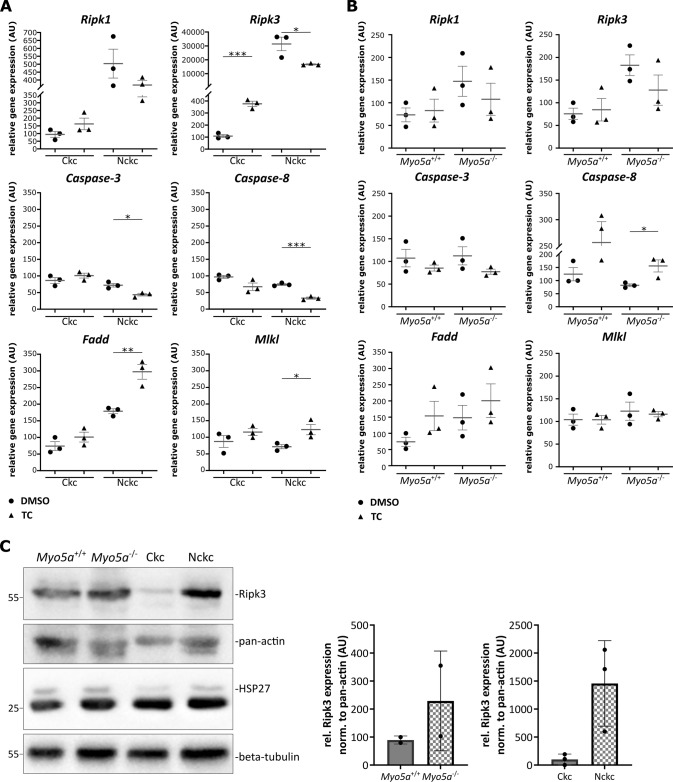
Increased expression of Ripk3 in cells lacking primary cilia. **A**, **B** Quantitative real-time PCR of several cell death-related genes in mIMCD3 cells revealed upregulation of necroptosis players in non-ciliated cells: **A** Ckc versus Nckc (*n* = 3); **B** control versus *Myo5a*^−/−^ (*n* = 3). Cells were treated with TNFα (TNF, 4 ng/100 µl) and cycloheximide (CHX, 2 µg/100 µl) for 16 h or with DMSO. Statistical analysis was performed by using a one-way ANOVA followed by a two-sided Student’s *t* test (*p* value: >0.001***; 0.002**; 0.033*; ns = 0.12). **C** Immunoblot analysis of lysates from untreated ciliated (*Myo5*^*+/+*^/Ckc) and non-ciliated (*Myo5a*^−/−^/Nckc) cells using Ripk3 (~57 kDa) and HSP27 (~27 kDa) antibodies. Pan-actin (~30 kDa) or beta-tubulin (~55 kDa) were used as controls.

To understand how the loss of primary cilia increases susceptibility to necroptotic cell death, we analyzed mRNA expression of cell death-related genes both in untreated and TC-treated cells, again comparing Ckc with Nckc (Fig. 3A) as well as Myo5a^+/+^ with Myo5a^−/−^ cells (Fig. 3B). While caspase-3 and caspase-8 expression levels were independent of the presence of primary cilia, these data revealed significantly higher expression levels of Ripk3 mRNA in non-ciliated cells and a trend toward increased expression for Ripk1 and Fadd. Immunoblotting confirmed increased levels of Ripk3 on the protein level in both cell lines without cilia (Fig. 3C). Susceptibility to TNFα could result from reduced NF-κB signaling in non-ciliated cells. However, immunoblots revealed increased levels of the NF-κB inhibitor IκBα in cells lacking cilia (Suppl. Fig. 3A, B), indicating increased NF-κB activity. Short-term stimulation with TNFα led to increased phosphorylation and consistently degradation of IκBα while inducing phosphorylation of NF-κB p65 at Ser536. Consistently, qPCR data on NF-κB expression (Suppl. Fig. 3C, D) confirmed that NF-κB activity is not reduced in non-ciliated cells. Therefore, the upregulation of Ripk3 and Ripk1 in non-ciliated cells can explain their marked susceptibility to necroptosis, and the underlying molecular mechanisms leading to Ripk3/Ripk1 upregulation remain elusive.

### Proteomic profiling identifies deregulation of autophagy-related and lysosomal proteins

**Fig. 4:**
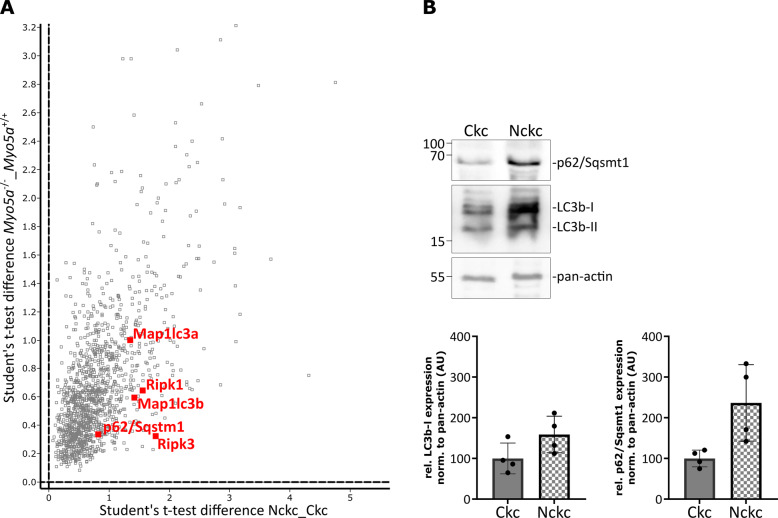
Loss of cilia induces upregulation of the p62/Ripk1 module. **A** Details from the scatter blot (total plot in Suppl. Fig. 4E) highlighting proteins connecting the autophagosome and the necrosome. **B** Immunoblot analyses of DMSO treated Nckc versus Ckc for LC3 (~17 kDa) and p62/Sqstm1 (~62 kDa) expression (*n* = 4) and densitometric analysis, normalized to pan-actin (*n* = 4).

To gain additional mechanistic insights into the increase in necroptotic death and in Ripk3 expression in non-ciliated cells, we performed an unbiased proteomic analysis to identify differentially expressed proteins and pathways related to the loss of cilia in the respective cell lines. Principal component analyses clearly separated CkC from Nckc (Suppl. Fig. 4A), as well as Myo5a^−/−^ from Myo5a^+/+^ control cells (Suppl. Fig. 4B). Compared to the respective controls and based on a Student’s *t*-test with standard parameter (S0 = 0 and threshold *p* value > 0.05), we found 3094 differentially expressed proteins in Nckc and 2980 differentially expressed proteins in Myo5a^−/−^ (Supplementary Table S2). The identified differentially expressed proteins were used as input for clustered heat maps of both datasets (Suppl. Fig. 4C, D). To identify the common proteins and pathways altered upon loss of cilia, we compared significantly regulated proteins from both datasets to visualize the similarities of both unciliated cell lines. Student’s t-test difference of non-ciliated cells versus ciliated cells correlates, which reveals 2282 equally upregulated and 832 down-regulated proteins demonstrating the similarity between the two loss-of-cilia models (Suppl. Fig. 4E). Gene ontology and KEGG pathway analyses of the clustered non-ciliated data set of significantly up or down-regulated proteins revealed terms related to spliceosome and lysosome to be enriched (Suppl. Fig. 5A). Indeed, many autophagy proteins were significantly altered in both unciliated cell lines, as shown in representative volcano blots (Suppl. Fig. 5B, C). Strikingly, we observed an enrichment of proteins previously shown to connect the autophagosome with the necrosome, particularly an increased expression of Map1lc3a/b (LC3) and p62/Sqsmt1, as well as Ripk1 and Ripk3 (Fig. 4A). This increased expression of LC3 and p62/Sqsmt1 in non-ciliated cells could be further confirmed by immunoblotting (Fig. 4B).

### Necroptosis in the kidney upon loss of cilia

**Fig. 5:**
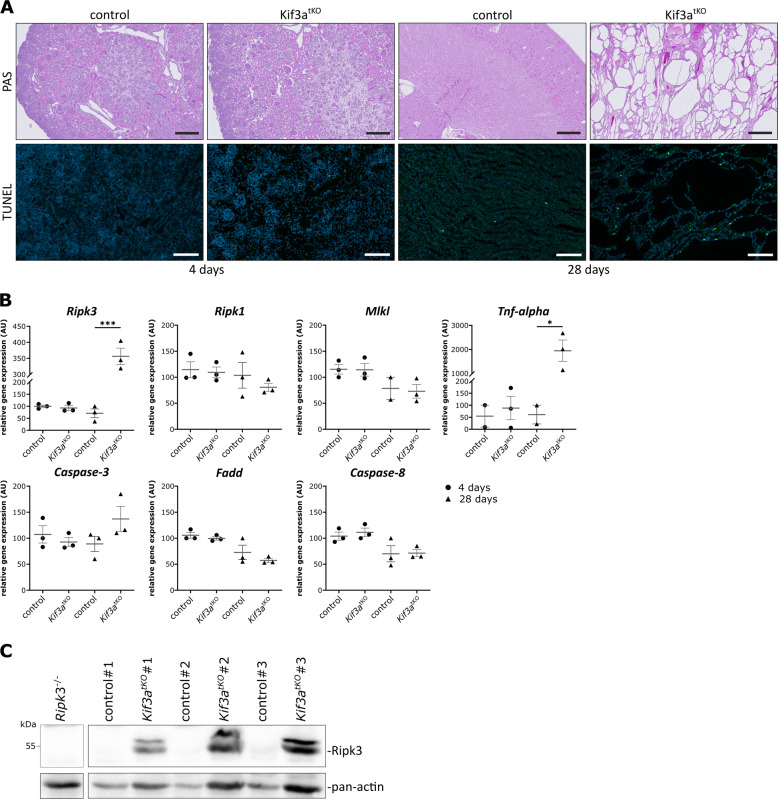
Genetic targeting of ciliogenesis leads to cell death and increased Ripk3 expression in vivo. **A** PAS staining of kidneys from *Kif3a*^fl/fl^:Ksp:cre^+/−^ and *Kif3*^fl/wt^:Ksp:cre^+/−^ mice at a postnatal age of 4 days (scale bar 200 µm) and 28 days (scale bar 500 µm) showing the loss of kidney architecture and cyst formation over time. TUNEL staining (scale bar 100 µm) indicates cell death. **B** Quantitative real-time PCR of several cell death genes showing upregulation of necroptosis-specific genes in mouse tissue lacking primary cilia (*n* = 3). Statistical analysis was performed by using a one-way ANOVA followed by a two-sided Student’s *t* test (*p* value: >0.001***; 0.002**; 0.033*; ns = 0.12). Control, heterozygous transgenic mice. **C** Immunoblot analysis of 28-day-old mice for Ripk3 expression (~57 kDa; *n* = 3 individual animals shown).

To understand the significance of our findings in vivo, we studied cell death in mice lacking functional primary cilia in the distal part of the nephron. Specifically, we knocked out the kinesin Kif3a in the distal tubules and collecting ducts of the kidney using the Ksp:Cre line. Kif3a is a subunit of the kinesin-2 motor required for intraflagellar transport, the transport of cargo along ciliary microtubules [39]. These mice develop cystic kidney disease starting with tubular dilatations in the first week of their life [40]. We used kidneys of Kif3a^fl/fl^:ksp:cre^+/−^ (Kif3a^tko^) and Kif3a^fl/wt^:ksp:cre^+/−^ (control) mice at postnatal days P4 and P28 and found TUNEL positivity increasing with age (Fig. 5A). Notably, qPCR analysis revealed a significant upregulation of Ripk3 in Kif3a^tko^ kidneys together with an increase in TNFα mRNA levels at P28, while caspase-3, caspase-8, Fadd, Mlkl, and Ripk1 were not significantly altered (Fig. 5B). Moreover, we detected high levels of Ripk3 in protein lysates from those kidneys, indicating an increased propensity to necroptosis during renal cyst formation and tissue degeneration (Fig. 5C). It is important to note that we did not detect any significant alterations in the expression of cell death genes at P4 at the time when kidney tissue showed almost no signs of cyst formation. However, cilia were described to be normal at birth (P0) in this mouse model [40], and cells of the distal nephron still carry some primary cilia at P4 (Suppl. Fig. 6, DBA-positive tubules). This is in line with the fact that loss of cilia itself is not sufficient to trigger cell death but can increase susceptibility to necroptosis under inflammatory conditions involving TNF or IFNγ. So far, we can only speculate about the factors that trigger necroptotic cell death during the early phase prior to cyst formation in Kif3a^tko^ mice.

### The loss of the ciliary signaling protein Nphp1 enhances necroptotic cell death

**Fig. 6:**
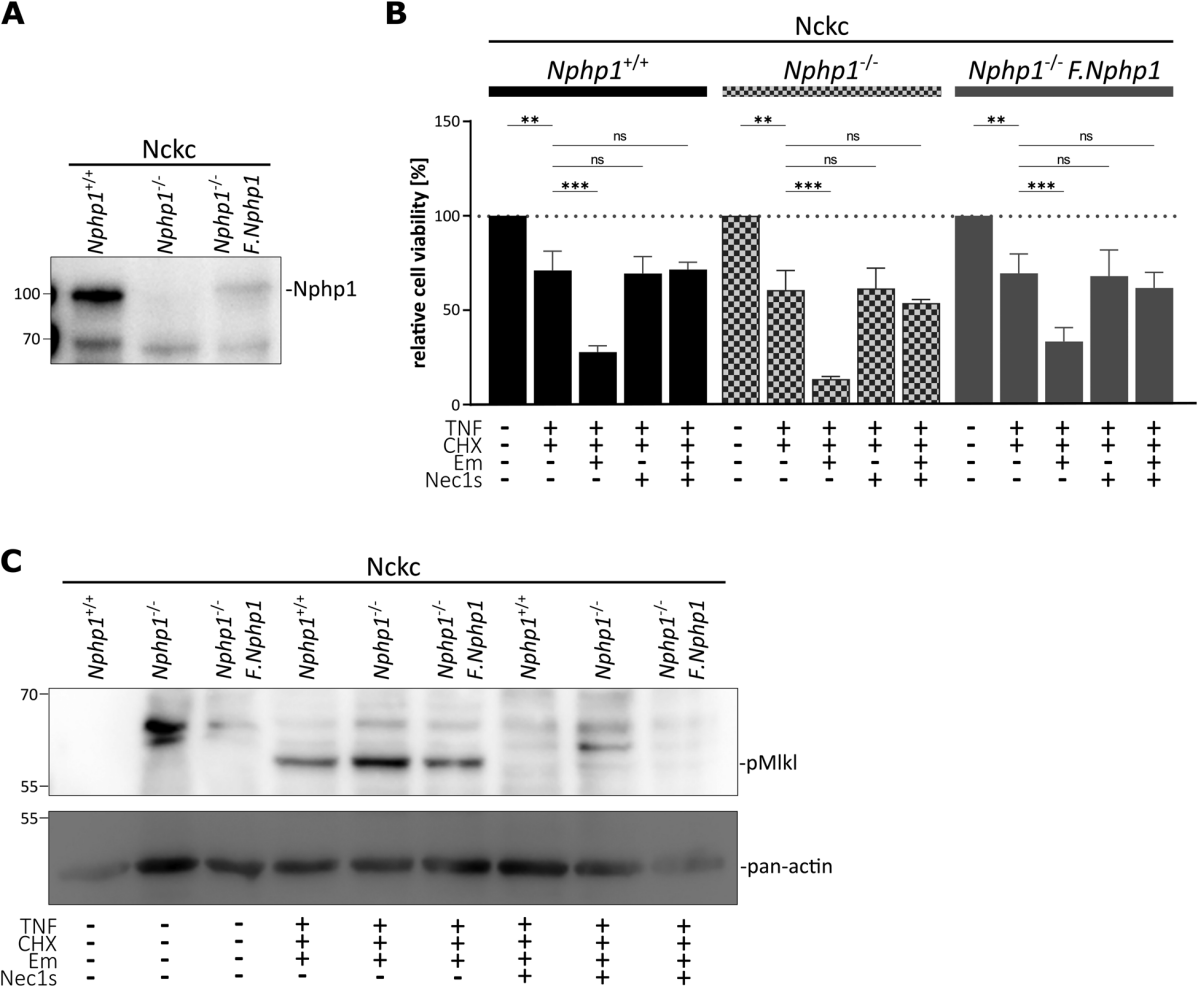
Loss of the functional but not structural ciliary protein Nphp1 enhances the necroptotic response. **A** Immunoblot analysis demonstrating Nphp1 deficiency in Nphp1^−/−^ cells and confirming re-expression of FLAG.Nphp1 by using Nphp1 (~83 kDa). **B** Neutral-red assay in Nckc proficient and deficient in Nphp1. RCD induction with TNFα (TNF, 4 ng/100 µl) and cycloheximide (CHX, 2 µg/100 µl) for 16 h. Additionally, caspase-8 inhibitor emricasan (Em, 10 µM) and necroptosis inhibitor necrostatin-1s (Nec1s, 40 µM) were used for 16 h (*n* = 3). Knockout of Nphp1 resulting in increased necroptotic death. **C** Immunoblot of phospho-Mlkl (~56 kDa) in Nphp1 proficient and deficient cells upon TC and TCE treatment for 8 h. Pan-actin was used as a loading control (*n* = 3).

Deletions of *NPHP1* are the most frequent cause of NPH, a pediatric ciliopathy and kidney disease that is characterized by tubular atrophy, cyst formation, interstitial fibrosis, and inflammation [11, 12, 41]. Nphp1 does not encode for a structural ciliary protein but for the key protein of the NPHP-protein complex involved in ciliary signaling [21]. Nphp1 is localized at the transition zone of primary cilia [42]. Therefore, Nphp1 deficiency does not result in the loss of primary cilia but rather causes cilia signaling defects. Kidneys have the capacity for intrinsic repair. Repair is based on dedifferentiation and proliferation of renal tubular cells without the need for prespecified stem cell populations and involves regulated disassembly and reassembly of primary cilia [43, 44], allowing the cells to reenter the cell cycle and undergo cell division [45]. Since the ciliary basal body, a modified centriole, is required to form the later spindle poles, cells have to disassemble the cilium prior to cell cycle re-entry. In this scenario, increased susceptibility to necroptosis might be of particular importance. To study whether loss of *Nphp1* might promote necroptotic damage under such conditions, we generated *Nphp1*^−/−^ cells in the non-ciliated Nckc subclone. As an additional control, we used single-copy integration into the *Rosa26* locus to re-express low levels of FLAG-tagged Nphp1. Expression of Nphp1/F.Nphp1 was controlled by immunoblotting of cell lysates using a specific Nphp1 monoclonal antibody [46] (Fig. 6A). When performing cell viability assays, we shortened the treatment time to 8 h to gain a larger number of surviving cells upon TCE treatment. The knockout of *Nphp1* indeed enhanced necroptotic response, which could be rescued by necrostatin-1s and partially by the re-expression of FLAG.Nphp1 (Fig. 6B). Immunoblotting again revealed the activation of Mlkl as indicated by phosphorylation (Fig. 6C). These data might indicate that in the absence of cilia Nphp1-related signaling is responsible for suppressing necroptosis.

## Discussion

Given the massive loss of tubular epithelial cells during the progression of renal ciliopathies, we investigated whether cilia could shape the response of renal epithelial cells upon induction of cell death. Interestingly, while the majority of ciliated renal epithelial cells underwent apoptosis after exposure to TNF and CHX, they did not appear to involve necroptosis as the inhibition of caspase activity did not induce necroptosis in these cells. Remarkably, this changed with the loss of primary cilia. In cells lacking cilia, apoptosis was reduced when exposed to TNF and CHX. Such protection from apoptosis might be important under physiological conditions when cells transiently disassemble their cilium prior to cell cycle re-entry and repair of tubular injuries. However, further inhibition of caspase activity in non-ciliated cells, mimicking inflammatory conditions, led to massive RIPK1-mediated necroptotic cell death, as indicated by the phosphorylation of MLKL. We can thus show for the first time that the absence of cilia switches the response of cells from apoptosis to necroptosis. Notably, the mere loss of cilia is not sufficient to drive cells into necroptosis. This is consistent with the phenotype of mice bearing genetic alterations that affect cilia or related human diseases since kidneys typically are unaffected at birth. Defective cilia, however, increase susceptibility to necroptosis under inflammatory conditions, and there must be additional factors in the progression of kidney disease that eventually initiate necroptotic cell death.

Mechanistically, we found Ripk3 to be upregulated both in mIMCD3 cell lines without cilia as well as in kidneys from mice lacking cilia in the distal tubules. We could detect both increased levels of mRNA expression as well as of Ripk3 protein. A number of factors have been recently described to modulate Ripk3 expression, which includes methylation of the Ripk3 promotor [47] and components of the NF-κB signaling pathway. It has recently been demonstrated that NF-κB1 and NF-kappa-B essential modulator (Nemo) bind to the *Ripk3* promotor and suppress TNFα-induced Ripk3 expression and necroptosis in endothelial cells [48]. Consistently, genetic inhibition of NF-kB signaling in the murine skin triggered TNFR1-mediated necroptosis and inflammation [49]. With respect to cilia, previous studies have found repression of NF-κB upon loss of cilia due to loss of Kif3a in hippocampal neurons [50] or due to hypomorphic *Ift88* mutation in chondrocytes [51]. The latter study suggested a crucial role of Hsp27 as a ciliary protein and known regulator of IKK [52–54]. Notably, we found no evidence for increased NF-κB signaling in mIMCD3 cells lacking cilia, as indicated by IκBα expression and its phosphorylation and degradation upon TNFα stimulation. Moreover, we found Hsp27 expression to be unaffected by loss of cilia (Fig. 3C). Therefore, the shift in cell death response toward necroptosis and the increase in Ripk3 might not be related to altered NF-κB activity.

Our unbiased approach provided additional mechanistic insights. Comparing protein expression of ciliated and non-ciliated cells followed by KEGG pathway analyses highlighted the enrichment of spliceosomal and lysosomal components in cells without cilia. The latter finding was surprising since the loss of cilia has been shown to negatively regulate autophagy [55–57]. Interestingly, the autophagy machinery is connected to the necrosome through p62/Sqsmt1 and Map1lc3a/b (LC3) interacting with Ripk1, and this interaction can control switching from apoptosis to necroptosis [58]. In particular, p62-mediated recruitment of Ripk1 to the autophagy machinery turns cell death from apoptosis toward necroptosis. Strikingly, our proteome data set demonstrates an increased abundance of p62/Sqsmt1, Map1lc3a/b (LC3), and Ripk1 in cells lacking primary cilia, which could be confirmed independently by immunoblots. In conclusion, the increased propensity to necroptosis upon loss of cilia might result from increased Ripk3 levels combined with a high abundance of the necrosome – autophagosome connecting module.

Loss of *NPHP1* is the most frequent genetic cause of pediatric cystic kidney diseases [41]. Here, we demonstrate that loss of *Nphp1* further promotes necroptosis in cells without cilia. As described above, loss of cilia occurs regularly in the kidney: the repair of tubular cellular damage requires surviving resident cells to disassemble the cilium prior to cell cycle re-entry [43, 44]. At this point, the increased susceptibilities to necroptosis due to the ciliopathy mutation on the one side and due to the missing cilium on the other side might add up in such a way that a critical threshold is exceeded and the necroptotic rate in the tissue increases. Given the increasing number of pharmacological interventions targeting different routes of cell death, including necroptosis [59, 60], it will be critical to analyze the specific role of this cilia cell-death switch in the pathogenesis of individual ciliopathies.

## Material and methods

### Cell lines and cell culture

Murine inner medullary collecting duct 3 cells (mIMCD3, ATCC CRL-2123™) [61], were cultured in DMEM-F12 medium (Sigma) supplemented with 10% fetal bovine serum (FBS, Gibco™), 2 mM GlutaMAX (Gibco™) and 1.0% Penicillin and Streptomycin (Gibco™). Cells were maintained at 37 °C in the presence of 5% CO_2_. All cell lines were tested negative for mycoplasma (PCR Mycoplasma Test Kit I/C, PromoKine). *Myo5a*^−/−^ mIMCD3 cells generated with CRISPR/Cas9 mediated genome editing has been described earlier [37]. mIMCD3 subclones (ciliated kidney cells (Ckc) and non-ciliated kidney cells (Nckc)) were generated by sorting single cells into a 96-well plate using a FACSAria*III*. After expansion, cell clones were screened for the number of ciliated cells using immunofluorescence stainings (acetylated tubulin/Arl13b). *Nphp1* deficient cells were generated based on Nckc’s using vector-based genome editing as described [37]. The sgRNA (5′-AGCGCCTGCAGCGGGTCCCG–CGG-3′) was cloned into PX458. pSpCas9(BB)‐2 A‐GFP (PX458), a kind gift from Feng Zhang (Addgene plasmid # 48138) [62].

### Live-cell Imaging

Myo5a^+/+^ and *Myo5a*^−/−^ mIMCD3 cells, as well as the mIMCD3 subclones Ckc and Nckc, were seeded, with 15,000 cells per well, into 96-well plates in triplicates. 24 h after seeding, cells were treated with DMSO (AppliChem), 4 ng/100 µl TNFα (aa80–235; R&D), 2 µg/100 µl cycloheximide (C4859; Sigma), and 10 µM emricasan (Em; SEL-S7775; Biozol). For the IFNγ stimulation experiments, 10,000 cells per well were seeded. On the following day, cells were preincubated with 1000 U/ml IFNγ (#315–05; PeproTech) for 8 h, before combined treatment with IFNγ and 10 µM Em. For both experiments, cell death was visualized by adding DiYO-1 (ABD-17580, Biomol). Immediately after adding the reagents, the plates were transferred to the IncuCyte® S3 (Sartorius; 37 °C and 5% CO_2_), and the first images were captured (T0). Subsequently, every 2 hours, pictures were taken. Per well, three single images were generated for each time point. In total, plates were scanned over the period of 24 h, thereby imaging the green channel with 300 ms exposure time and the phase contrast channel with ×20 objective. The analysis was done by teaching the machine for positive events within the included IncuCyte® Cell-by-Cell Analysis Software Module (#9600–0031, Sartorius). For analysis, a multiple comparison one-way ANOVA was performed, using the Turkey test with *p* < 0.05.

### Immunofluorescence staining

mIMCD3 cells were seeded on coverslips to stain for primary cilia [37]. Cells were fixed with 4% PFA for 5 min at RT followed by 4 min incubation with ice-cold methanol at −20 °C. Next, cells were incubated with blocking solution 1xPBS containing 0.1% Triton X-100 and 10% normal donkey serum (Jackson ImmunoResearch) for 1 h at RT, followed by an 80 min incubation at RT with primary antibody (anti-acetylated tubulin, T6793 Sigma, 1:1000; anti-Arl13B, 17711–1-AP ProteinTech, 1:400). The following secondary antibodies were used: donkey-anti-rabbit Cy3, 715-165–150, and donkey-anti-mouse-Alexa 488, 715–545–150; both Jackson ImmunoResearch, 1:500; for 45 min at RT. Samples were mounted in ProLong™ Diamond with DAPI (ThermoFisher Scientific). Kidney tissue staining of 4 µm fixed sections were performed as previously described [63]. Firstly, the sections were deparaffinized by xylene treatment followed by rehydration in graded ethanol (70%, 95%, 100%). Antigen retrieval was achieved using heat-induced epitope retrieval and citrate buffer. For immunohistochemical staining, endogenous peroxidases and unspecific antibody binding sites were blocked by incubating with 1% BSA and 5% donkey serum (Jackson ImmunoResearch) for 1 h at RT. The primary antibody (anti-acetylated Tubulin, T6793 Sigma, 1:1000) was incubated overnight at 4 °C in the blocking solution, followed by incubation with fluorophore-coupled secondary antibody anti-mouse-Cy5, # 715–175–150, Jackson ImmunoResearch, 1:500) or tubule markers (Rodamin-DBA (RL-1032–2); FITC-Lotus Tetragonolobus Lectin (LTL, FL-1321–2; Vector laboratories) both 1:500) for 1 h at RT. The samples were mounted after a short pre-incubation of Hoechst33342 (ThermoFisher Scientific, 1:1000) with ProLong™ Diamond (ThermoFisher Scientific). Images were acquired using the AxioObserver microscope with an axioCam ICc 1, Axiocam 702 mono, Apotome system (Carl Zeiss MicroImaging, Jena, Germany; objectives Plan-Apochromat 20x/0.8 and EC Plan-Neofluar 40x/1.3).

### Cell viability assay

Neutral-red release (NR) assays for cell viability were performed as described [64]. In brief, 30,000 cells were seeded as triplicates in 96-well plates 24 h prior to treatments. Cells were treated with DMSO (AppliChem), 4 ng/100 µl TNFα (aa80–235; R&D), 2 µg/100 µl cycloheximide (C4859; Sigma), 5 µM. birinapant (SELS7015, Biozol), 10 µM emricasan (SEL-S7775; Biozol), 40 µM Necrostatin-1s (ab221984; Abcam), and 5 µM GSK872 (HY-101872, Sigma) as indicated in the figures and incubated for 16 h at 37 °C. Once 14 h of treatment was passed, neutral-red (C.I.50040, Sigma) was added to the medium. After additional 2 h, the cells were washed thrice with PBS followed by a 15 min incubation of destaining buffer (50% EtOH, 49% ddH_2_O, and 1% acetic acid) under gentle shaking [65]. The absorbance was measured at 540 nm using the Infinite® M Plex plate reader (TECAN).

### Mouse lines

To generate mice lacking primary cilia in the distal tubules and collecting ducts of the kidneys, *Kif3a*^fl^ mice [40] were crossed with Ksp:cre [66] mice on a C57Bl/6 N background. The mice were housed according to standardized specific pathogen-free conditions in the in vivo research facility of CECAD at the University of Cologne. All matings and experiments were conducted in accordance with European, national and institutional guidelines, as approved by the State Office of North Rhine-Westphalia, Department of Nature, Environment and Consumer Protection (8.87-50.10.31.08.049 and 84–02.04.2013.A152). For the preparation of the mice, the mice were sacrificed by cervical dislocation, and kidneys were perfused with PBS through the aorta. Tissue was processed by fixation in 4% formaldehyde and embedding in paraffin as well as snap-frozen for further tissue analysis.

### Immunohistology and TUNEL staining

For histological analysis, tissue was cut into 1-μm-thick sections and deparaffinized by xylene treatment and rehydration in graded ethanol. Sections were stained with 0.9% periodic acid (cat# 3257.1, Roth) and Schiffsches Reagent (cat#1.09033, Merck) both for 10 min embedded into washing steps with H_2_O. Finally, to visualize nuclei in blue, the samples were stained with Mayer’s Haematoxylin for 20 s. After dehydration of the sections, they were embedded with Histomount (HS-103, National Diagnostics). The DeadEnd™ Fluorometric TUNEL System (Promega) was performed following the manufacturer’s instructions, with the exception that the samples were mounted, with a pre-incubation of Hoechst (ThermoFisher Scientific, 1:1000) as nuclear staining, with ProLong™ Diamond (ThermoFisher Scientific). The antibody signals were visualized by using the Axio Observer as described above.

### Immunoblotting

mIMCD3 cells were seeded in six-wells plates/dishes and treated with DMSO, TNFα, CHX, Nec1s, or emricasan as described above for 16 h. For whole-cell extracts for pMlkl analysis, cells were immediately lysed in 1× Laemmli buffer. For protein lysates, cells were harvested in medium and, after centrifugation, lysed in RIPA buffer (1% IgePAL, 150 mM NaCl, 0.25% Na-Deoxy, 50 mM Tris pH 7.5) supplemented with cOmplete™ Protease Inhibitor Cocktail (Roche). For immunoblotting of kidney samples, 30 mg of tissue were homogenized with a Wheaton Dounce tissue grinder in RIPA buffer on ice. After 30 min on ice, lysates were centrifuged at 14,000 rpm for 30 min at 4 °C. Protein concentration was measured from the supernatants using Pierce BCA Protein Assay Kit (ThermoFisher Scientific) according to the manufacturer’s instructions. Finally, samples were diluted with 5x sample buffer. Proteins were separated by SDS–PAGE and transferred to a PVDF-FL membrane (Millipore) and, after blocking with Intercept blocking solution (Licor) and washing (1× PBS, 0.1% Tween-20), stained with antibodies against phospho-Mlkl Ser345 (#37333, CST, 1:1000), Ripk1 (#610459, bd biosciences, 1:1000), Ripk3 (ADI-905–242, Enzo, 1:1000), cleaved-caspase-3 Asp175 (#9661, CST, 1:1000), LC3 (#2775 S, CST, 1:1000), p62/Sqstm1 (GP62-C, Progen, 1:1000), total IκBa (sc-371, Santa Cruz, 1:1000), pIκBa (#9246, CST, 1:1000), pNFκB (#3033, CST, 1:1000), Nphp1 (Homemade polyclonal rabbit, 1:1000), β-Tubulin (E7, DSHB, 1:500) or pan-actin (#8456, CST, 1:1000) at 4 °C overnight. Fluorescence-coupled secondary antibodies (anti-mouse IgG (H + L) IRDye 680RD, cat# 926–68070; anti-rabbit IgG (H + L) IRDye 680RD, cat# 926–68071; anti-mouse IgG (H + L) IRDye 800CW, cat# 926–32210; anti-rabbit IgG (H + L) IRDye 800CW, cat# 926–32211; Licor) were incubated for 45 min at RT. Finally, the membranes were scanned using Odyssey CLx (Licor). Densitometry was performed by using ImageJ, normalized to the housekeeping protein, and statistically analyzed with a two-tailed Student’s *t* test; *p* < 0.05.

### Mass spectrometry

For each of the four biological replicates per point, one 10 cm dish of mIMCD3 cells of the indicated genotype was harvested and snap-frozen. Pellets were resuspended in urea buffer (8 M Urea, 50 mM ammonium bicarbonate) containing Halt protease-phosphatase-inhibitor cocktail (Thermo Scientific). After clearing of the sample (16,000 × *g*, 1 h at 4 °C), the lysates were reduced (10 mM dithiothreitol, 1 h, at RT) and alkylated (50 mM chloroacetamide, 1 h, at RT). Samples were diluted to 2 M urea and subjected to tryptic digestion (enzyme:substrate ratio of 1:50). After overnight incubation, a double-layered stage-tip clean-up (C18) was performed. Samples were handed in for analysis into two separated experiments: Nckc versus Ckc and Myo5a^−/−^ versus Myo5a^+/+^ control cells. Samples were analyzed at the CECAD proteomics facility on an Orbitrap Exploris 480 (Thermo Scientific) mass spectrometer equipped with a FAIMSpro differential ion mobility device coupled to an UltiMate 3000 (Thermo Scientific). LFQ values were calculated using the DIA-NN R-package [67]. A Swissprot mouse canonical database (UP589, downloaded 18/06/20) was used for library building with settings matching acquisition parameters and the match-between-runs function enabled. Here, samples are directly used to refine the library for a second search of the sample data. DIA-NN was run with the additional command-line prompts “—report-lib-info” and “—relaxed-prot-inf”. Further output settings were: filtered at 0.01 FDR, N-terminal methionine excision enabled, maximum number of missed cleavages set to 1, min peptide length set to 7, max peptide length set to 30, min precursor m/z set to 400, max precursor m/z set to 1000, cysteine carbamidomethylation enabled as a fixed modification. Afterward, DIA-NN output was further filtered on library q-value and global *q* value < = 0.01 and at least two identified peptides per protein using R (4.1.3). Student’s t-tests and Fisher exact tests were calculated in Perseus (version 1.6.15.0) after the removal of decoys and potential contaminants [68]. Data were filtered for at least four out of four values in at least one condition. The remaining missing values were imputed with random values from a normal distribution using Perseus defaults. The mass spectrometry proteomics data have been deposited to the ProteomeXchange Consortium via the PRIDE [69] partner repository with the data set identifier PXD035290.

### Quantitative real-time PCR

mIMCD3 cells were seeded in 12 well plates, treated with DMSO (AppliChem), 4 ng/100 µl TNFα (aa80–235; R&D), 2 µg/100 µl cycloheximide (C4859; Sigma) for 16 h and washed with PBS right before lysis in Tri-Reagent (Sigma). For RNA isolation from kidney tissue, one-quarter of a kidney was ground with BeadBeater (Roth) using a Precelly24 with 5000 rpm two times for 30 s in Tri-Reagent. RNA extraction was performed with the Direct-zol RNA Miniprep kit (Zymo Research) following the manufacturer’s instructions, including a DNase1 treatment step. Prior to the reverse transcription by using the High-Capacity cDNA Reverse Transcription kit (Applied Biosystems), RNA concentration and sample quality were assessed on a Nanodrop spectrophotometer (Peqlab). mRNA was assessed by SYBR Green (ThermoFisher Scientific) qPCR using mHprt1 as endogenous control. Primers are listed in Supplementary Table S1. The qPCR experiments were performed on a QuantStudio 12 K Flex Real-time PCR System (ThermoFisher Scientific). For data analysis, all results were normalized to the housekeeping gene *Hphrt1* using the delta-delta CT followed by a two-tailed Student’s *t* test (*p* < 0.05).

### Quantification and statistical analysis

Data are expressed as mean ± standard deviation (SD). All experiments were performed in at least three independent biological replicates. The data were statistically analyzed with GraphPad Prism version 8.0.2.

## Supplementary information

Suppl. Fig. 1

Suppl. Fig. 2

Suppl. Fig. 3

Suppl. Fig. 4

Suppl. Fig. 5

Suppl. Fig. 6

Suppl. Fig. 7: original data file

Suppl. Table 1

Suppl. Table 2

## Data Availability

The mass spectrometry proteomics data have been deposited to the ProteomeXchange Consortium via the PRIDE [69] partner repository with the data set identifier PXD035290. All additional data generated or analyzed during this study are included in the article.

## References

[1] Wheway G, Nazlamova L, Hancock JT. Signaling through the primary cilium. Front Cell Dev Biol. 2018,6:8. 10.3389/fcell.2018.00008 PMC5809511 29473038

[2] Gerdes JM, Davis EE, Katsanis N (2009). The vertebrate primary cilium in development, homeostasis, and disease. Cell.

[3] Malicki JJ, Johnson CA (2017). The cilium: cellular antenna and central processing unit. Trends Cell Biol.

[4] Rohatgi R, Snell WJ (2010). The ciliary membrane. Curr Opin Cell Biol.

[5] Garcia-Gonzalo FR, Reiter JF. Open sesame: how transition fibers and the transition zone control ciliary composition. Cold Spring Harb Perspect Biol. 2017;9:a028134. 10.1101/cshperspect.a028134 PMC5287074 27770015

[6] Hildebrandt F, Benzing T, Katsanis N (2011). Ciliopathies. N. Engl J Med.

[7] Reiter JF, Leroux MR (2017). Genes and molecular pathways underpinning ciliopathies. Nat Rev Mol Cell Biol.

[8] McConnachie DJ, Stow JL, Mallett AJ (2021). Ciliopathies and the kidney: a review. Am J Kidney Dis.

[9] Bergmann C, Guay-Woodford LM, Harris PC, Horie S, Peters DJM, Torres VE (2018). Polycystic kidney disease. Nat Rev Dis Prim.

[10] Stokman MF, Saunier S, Benmerah A. Renal ciliopathies: sorting out therapeutic approaches for nephronophthisis. Front Cell Dev Biol. 2021;13:653138. 10.3389/fcell.2021.653138 PMC8155538 34055783

[11] Bollée G, Fakhouri F, Karras A, Noël L-H, Salomon R, Servais A (2006). Nephronophthisis related to homozygous NPHP1 gene deletion as a cause of chronic renal failure in adults. Nephrol Dialysis Transplant.

[12] Srivastava S, Molinari E, Raman S, Sayer JA (2017). Many genes-one disease? genetics of nephronophthisis (NPHP) and NPHP-associated disorders. Front pediatrics.

[13] Woo D (1995). Apoptosis and loss of renal tissue in polycystic kidney diseases. N. Engl J Med.

[14] Peintner L, Borner C (2017). Role of apoptosis in the development of autosomal dominant polycystic kidney disease (ADPKD). Cell Tissue Res.

[15] Forschbach V, Goppelt-Struebe M, Kunzelmann K, Schreiber R, Piedagnel R, Kraus A (2015). Anoctamin 6 is localized in the primary cilium of renal tubular cells and is involved in apoptosis-dependent cyst lumen formation. Cell Death Dis.

[16] Hopp K, Ward CJ, Hommerding CJ, Nasr SH, Tuan HF, Gainullin VG (2012). Functional polycystin-1 dosage governs autosomal dominant polycystic kidney disease severity. J Clin Invest.

[17] Ma M, Tian X, Igarashi P, Pazour GJ, Somlo S (2013). Loss of cilia suppresses cyst growth in genetic models of autosomal dominant polycystic kidney disease. Nat Genet.

[18] Shibazaki S, Yu Z, Nishio S, Tian X, Thomson RB, Mitobe M (2008). Cyst formation and activation of the extracellular regulated kinase pathway after kidney specific inactivation of Pkd1. Hum Mol Genet.

[19] Ghosh AK, Hurd T, Hildebrandt F (2012). 3D spheroid defects in NPHP knockdown cells are rescued by the somatostatin receptor agonist octreotide. Am J Physiol Ren Physiol.

[20] Delous M, Hellman NE, Gaudé H-M, Silbermann F, Le Bivic A, Salomon R (2009). Nephrocystin-1 and nephrocystin-4 are required for epithelial morphogenesis and associate with PALS1/PATJ and Par6. Hum Mol Genet.

[21] Sang L, Miller JJ, Corbit KC, Giles RH, Brauer MJ, Otto EA (2011). Mapping the NPHP-JBTS-MKS protein network reveals ciliopathy disease genes and pathways. Cell.

[22] Giles RH, Ajzenberg H, Jackson PK (2014). 3D spheroid model of mIMCD3 cells for studying ciliopathies and renal epithelial disorders. Nat Protoc.

[23] Vanden Berghe T, Linkermann A, Jouan-Lanhouet S, Walczak H, Vandenabeele P (2014). Regulated necrosis: the expanding network of non-apoptotic cell death pathways. Nat Rev Mol Cell Biol.

[24] Bertheloot D, Latz E, Franklin BS (2021). Necroptosis, pyroptosis and apoptosis: an intricate game of cell death. Cell Mol Immunol.

[25] Maremonti F, Meyer C, Linkermann A (2022). Mechanisms and models of kidney tubular necrosis and nephron loss. J Am Soc Nephrol.

[26] Holler N, Zaru R, Micheau O, Thome M, Attinger A, Valitutti S (2000). Fas triggers an alternative, caspase-8-independent cell death pathway using the kinase RIP as effector molecule. Nat Immunol.

[27] Grootjans S, Vanden Berghe T, Vandenabeele P (2017). Initiation and execution mechanisms of necroptosis: an overview. Cell Death Differ.

[28] Liu Y, Liu T, Lei T, Zhang D, Du S, Girani L (2019). RIP1/RIP3-regulated necroptosis as a target for multifaceted disease therapy (Review). Int J Mol Med.

[29] Kondylis V, Kumari S, Vlantis K, Pasparakis M (2017). The interplay of IKK, NF-κB and RIPK1 signaling in the regulation of cell death, tissue homeostasis and inflammation. Immunol Rev.

[30] Flores-Romero H, Ros U, Garcia-Saez AJ (2020). Pore formation in regulated cell death. EMBO J.

[31] Linkermann A, Brasen JH, Himmerkus N, Liu S, Huber TB, Kunzendorf U (2012). Rip1 (receptor-interacting protein kinase 1) mediates necroptosis and contributes to renal ischemia/reperfusion injury. Kidney Int.

[32] Linkermann A, Heller JO, Prokai A, Weinberg JM, De Zen F, Himmerkus N (2013). The RIP1-kinase inhibitor necrostatin-1 prevents osmotic nephrosis and contrast-induced AKI in mice. J Am Soc Nephrol.

[33] Newton K, Dugger DL, Maltzman A, Greve JM, Hedehus M, Martin-McNulty B (2016). RIPK3 deficiency or catalytically inactive RIPK1 provides greater benefit than MLKL deficiency in mouse models of inflammation and tissue injury. Cell Death Differ.

[34] Linkermann A, Skouta R, Himmerkus N, Mulay SR, Dewitz C, De Zen F (2014). Synchronized renal tubular cell death involves ferroptosis. Proc Natl Acad Sci USA.

[35] Mc Fie M, Koneva L, Collins I, Coveney CR, Clube AM, Chanalaris A, et al. Ciliary proteins specify the cell inflammatory response by tuning NFκB signalling, independently of primary cilia. J Cell Sci 2020;133:jcs239871. 10.1242/jcs.239871 PMC7358134 32503942

[36] Brumatti G, Ma C, Lalaoui N, Nguyen N-Y, Navarro M, Tanzer MC (2016). The caspase-8 inhibitor emricasan combines with the SMAC mimetic birinapant to induce necroptosis and treat acute myeloid leukemia. Sci Transl Med.

[37] Kohli P, Hohne M, Jungst C, Bertsch S, Ebert LK, Schauss AC (2017). The ciliary membrane-associated proteome reveals actin-binding proteins as key components of cilia. EMBO Rep.

[38] Wu CT, Chen HY, Tang TK (2018). Myosin-Va is required for preciliary vesicle transportation to the mother centriole during ciliogenesis. Nat Cell Biol.

[39] Marszalek JR, Ruiz-Lozano P, Roberts E, Chien KR, Goldstein LS (1999). Situs inversus and embryonic ciliary morphogenesis defects in mouse mutants lacking the KIF3A subunit of kinesin-II. Proc Natl Acad Sci USA.

[40] Lin F, Hiesberger T, Cordes K, Sinclair AM, Goldstein LS, Somlo S (2003). Kidney-specific inactivation of the KIF3A subunit of kinesin-II inhibits renal ciliogenesis and produces polycystic kidney disease. Proc Natl Acad Sci USA.

[41] Halbritter J, Porath JD, Diaz KA, Braun DA, Kohl S, Chaki M (2013). Identification of 99 novel mutations in a worldwide cohort of 1,056 patients with a nephronophthisis-related ciliopathy. Hum Genet.

[42] Schermer B, Hopker K, Omran H, Ghenoiu C, Fliegauf M, Fekete A (2005). Phosphorylation by casein kinase 2 induces PACS-1 binding of nephrocystin and targeting to cilia. EMBO J.

[43] Humphreys BD, Czerniak S, DiRocco DP, Hasnain W, Cheema R, Bonventre JV (2011). Repair of injured proximal tubule does not involve specialized progenitors. Proc Natl Acad Sci USA.

[44] Kusaba T, Lalli M, Kramann R, Kobayashi A, Humphreys BD (2014). Differentiated kidney epithelial cells repair injured proximal tubule. Proc Natl Acad Sci.

[45] Kim S, Tsiokas L (2011). Cilia and cell cycle re-entry: more than a coincidence. Cell Cycle.

[46] Liebau MC, Hopker K, Muller RU, Schmedding I, Zank S, Schairer B (2011). Nephrocystin-4 regulates Pyk2-induced tyrosine phosphorylation of nephrocystin-1 to control targeting to monocilia. J Biol Chem.

[47] Koo G-B, Morgan MJ, Lee D-G, Kim W-J, Yoon J-H, Koo JS (2015). Methylation-dependent loss of RIP3 expression in cancer represses programmed necrosis in response to chemotherapeutics. Cell Res.

[48] Gao S, Menendez M, Kurylowicz K, Griffin CT (2021). Genomic locus proteomic screening identifies the NF-κB signaling pathway components NFκB1 and IKBKG as transcriptional regulators of Ripk3 in endothelial cells. PLoS One.

[49] Kumari S, Van T-M, Preukschat D, Schuenke H, Basic M, Bleich A (2021). NF-κB inhibition in keratinocytes causes RIPK1-mediated necroptosis and skin inflammation. Life Sci Alliance.

[50] Baek H, Shin HJ, Kim J-J, Shin N, Kim S, Yi M-H (2017). Primary cilia modulate TLR4-mediated inflammatory responses in hippocampal neurons. J Neuroinflammation.

[51] Wann AK, Chapple JP, Knight MM (2014). The primary cilium influences interleukin-1beta-induced NFkappaB signalling by regulating IKK activity. Cell Signal.

[52] Dodd SL, Hain B, Senf SM, Judge AR (2009). Hsp27 inhibits IKKbeta-induced NF-kappaB activity and skeletal muscle atrophy. FASEB J.

[53] Parcellier A, Schmitt E, Gurbuxani S, Seigneurin-Berny D, Pance A, Chantôme A (2003). HSP27 is a ubiquitin-binding protein involved in I-kappaBalpha proteasomal degradation. Mol Cell Biol.

[54] Park KJ, Gaynor RB, Kwak YT (2003). Heat shock protein 27 association with the I kappa B kinase complex regulates tumor necrosis factor alpha-induced NF-kappa B activation. J Biol Chem.

[55] Morleo M, Franco B (2019). The autophagy-cilia axis: an intricate relationship. Cells.

[56] Pampliega O, Orhon I, Patel B, Sridhar S, Díaz-Carretero A, Beau I (2013). Functional interaction between autophagy and ciliogenesis. Nature.

[57] Wang S, Livingston MJ, Su Y, Dong Z (2015). Reciprocal regulation of cilia and autophagy via the MTOR and proteasome pathways. Autophagy.

[58] Goodall ML, Fitzwalter BE, Zahedi S, Wu M, Rodriguez D, Mulcahy-Levy JM (2016). The autophagy machinery controls cell death switching between apoptosis and necroptosis. Dev Cell.

[59] Kist M, Vucic D (2021). Cell death pathways: intricate connections and disease implications. EMBO J.

[60] Lou J, Zhou Y, Feng Z, Ma M, Yao Y, Wang Y, et al. Caspase-independent regulated necrosis pathways as potential targets in cancer management. Front Oncol. 2021;10:616952. 10.3389/fonc.2020.616952 PMC7921719 33665167

[61] Rauchman MI, Nigam SK, Delpire E, Gullans SR (1993). An osmotically tolerant inner medullary collecting duct cell line from an SV40 transgenic mouse. Am J Physiol.

[62] Ran FA, Hsu PD, Wright J, Agarwala V, Scott DA, Zhang F (2013). Genome engineering using the CRISPR-Cas9 system. Nat Protoc.

[63] Dafinger C, Rinschen MM, Borgal L, Ehrenberg C, Basten SG, Franke M (2018). Targeted deletion of the AAA-ATPase Ruvbl1 in mice disrupts ciliary integrity and causes renal disease and hydrocephalus. Exp Mol Med.

[64] Reader SJ, Blackwell V, O’Hara R, Clothier RH, Griffin G, Balls M (1989). A vital dye release method for assessing the short-term cytotoxic effects of chemicals and formulations. Altern. Lab Anim.

[65] Fritsch M, Günther SD, Schwarzer R, Albert MC, Schorn F, Werthenbach JP (2019). Caspase-8 is the molecular switch for apoptosis, necroptosis and pyroptosis. Nature.

[66] Shao X, Somlo S, Igarashi P (2002). Epithelial-specific Cre/lox recombination in the developing kidney and genitourinary tract. J Am Soc Nephrol.

[67] Demichev V, Messner CB, Vernardis SI, Lilley KS, Ralser M (2020). DIA-NN: neural networks and interference correction enable deep proteome coverage in high throughput. Nat Methods.

[68] Tyanova S, Temu T, Sinitcyn P, Carlson A, Hein MY, Geiger T (2016). The Perseus computational platform for comprehensive analysis of (prote)omics data. Nat Methods.

[69] Perez-Riverol Y, Bai J, Bandla C, Garcia-Seisdedos D, Hewapathirana S, Kamatchinathan S (2022). The PRIDE database resources in 2022: a hub for mass spectrometry-based proteomics evidences. Nucleic Acids Res.

