# Supplementary material for: Primary cilia suppress Ripk3-mediated necroptosis
Source: Cell Death Discov. 2022 Dec 2;8:477. doi: 10.1038/s41420-022-01272-2 (PMC9718801; doi:10.1038/s41420-022-01272-2)
Supplement: Supplementary file 1 — Suppl. Fig. 1 [file 41420_2022_1272_MOESM1_ESM.pdf]

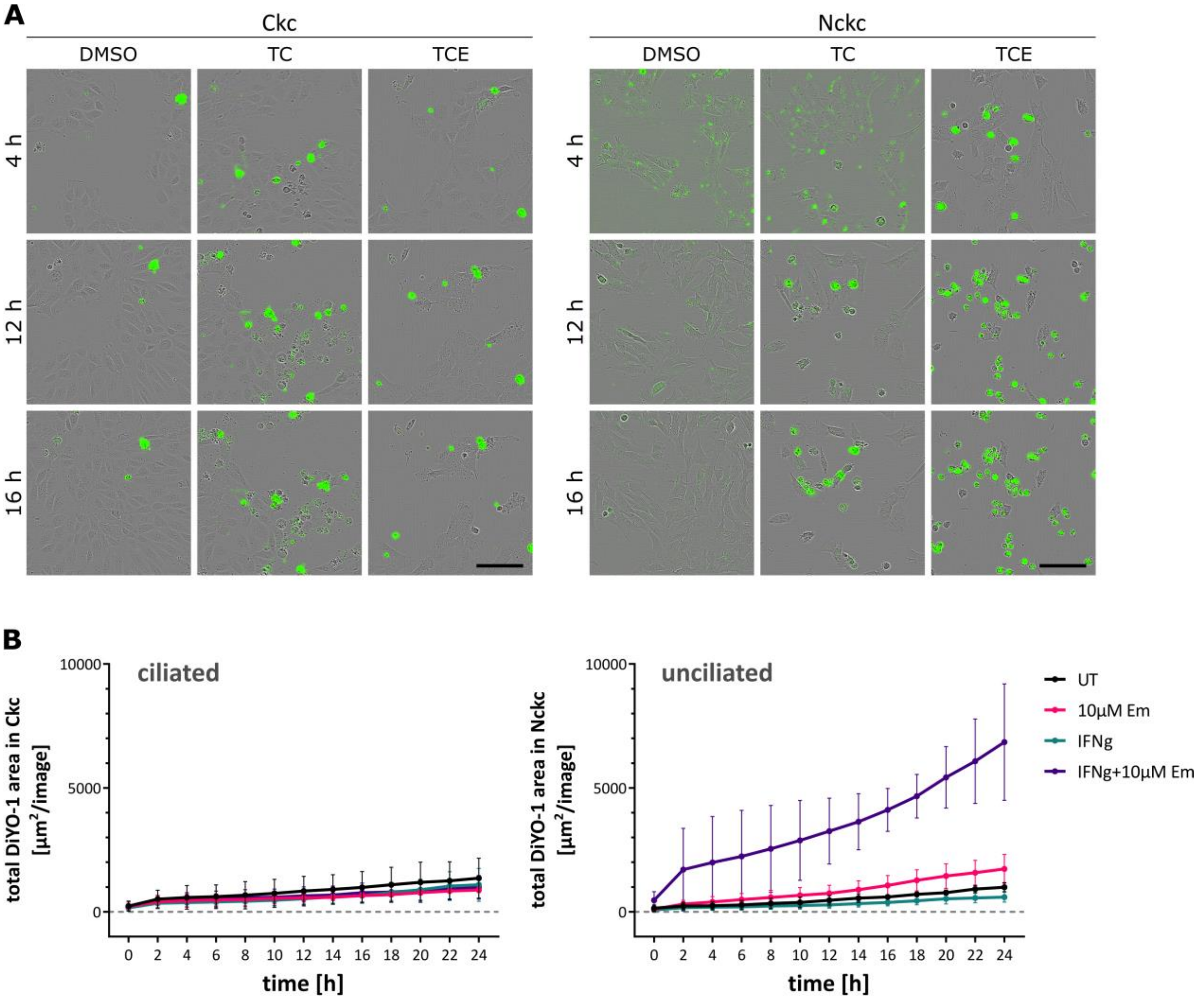

Supplementary figure 1

Suppl. Fig. 1: Necroptosis in non-ciliated kidney cells upon TCE and IFNγ/Emricasan treatment

(A) Representative pictures of cells from (Fig. 1 D). **(B)** Live-cell imaging over the period of 24 h after prestimulation with 1000 U/ml IFNγ for 8 h, and treatment with IFNγ and 10 µM Em or DMSO as control. Dead cells were stained with DiYO-1. Images were captured every 2 h (Ckc n=3; Nckc n=4).
