# Supplementary material for: Primary cilia suppress Ripk3-mediated necroptosis
Source: Cell Death Discov. 2022 Dec 2;8:477. doi: 10.1038/s41420-022-01272-2 (PMC9718801; doi:10.1038/s41420-022-01272-2)
Supplement: Supplementary file 2 — Suppl. Fig. 2 [file 41420_2022_1272_MOESM2_ESM.pdf]

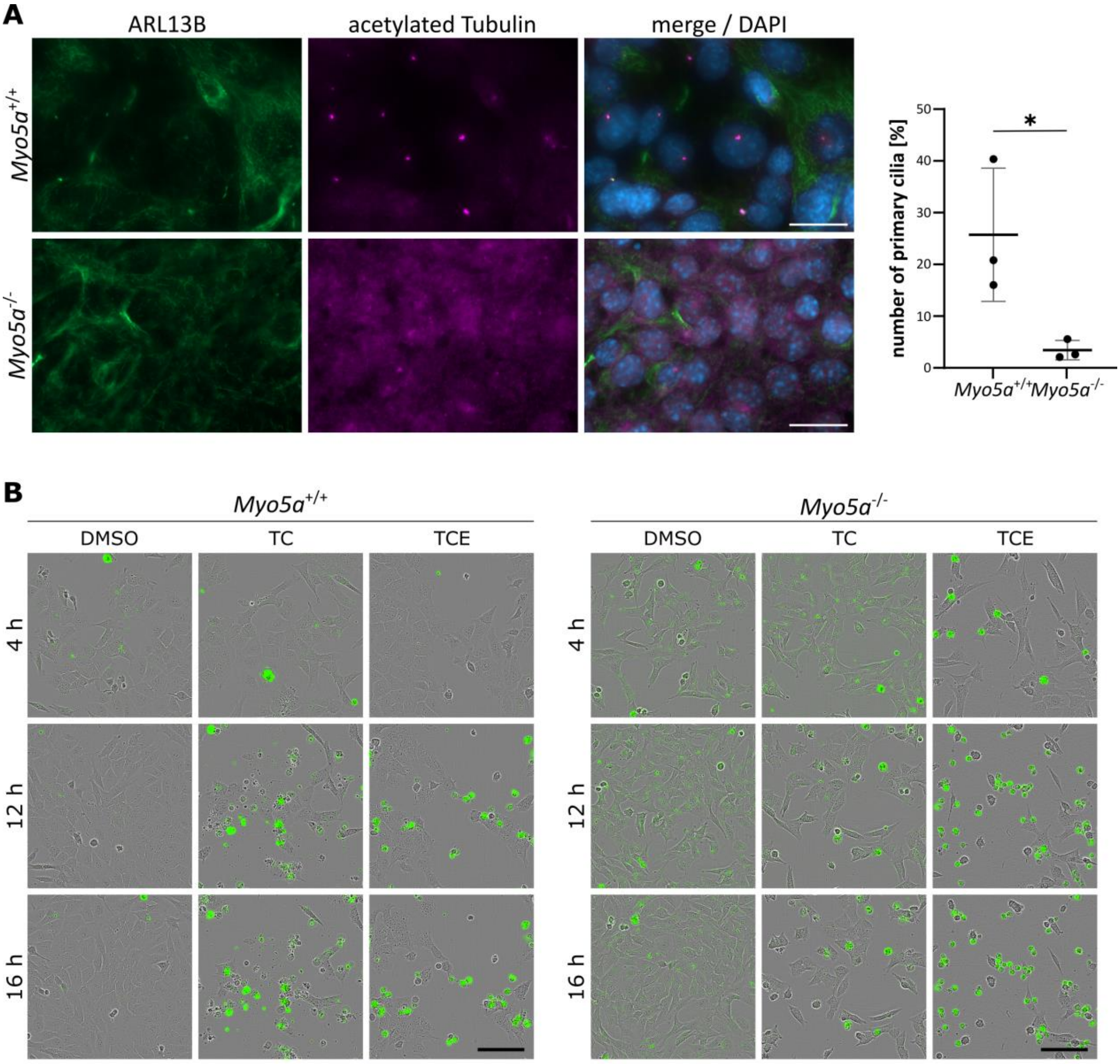

Supplementary figure 2

Suppl. Fig. 2: Loss of ciliogenesis and necroptosis susceptibility in Myo5a$^{-/-}$ cells

(A) Immunofluorescence of primary cilia in *Myo5a*$^{+/+}$ and *Myo5a*$^{-/-}$ cells (ARL13B (magenta), acetylated tubulin (green) and nuclei marker DAPI (blue); scale bar 20 µm). Quantification of primary cilia (n=3; total count of 924 cells for control and 875 cells for *Myo5a*$^{-/-}$). **(B)** Live-cell imaging of control and *Myo5a*$^{-/-}$ cells, over the period of 24 h, beginning with treatment induction of TNF, CHX (TC) and Em (TCE) or either DMSO as control. Cells were stained with the dead cell marker DiYO-1 (green). Images were captured every 2 h with a 20x objective (n=8).
