# Supplementary material for: Primary cilia suppress Ripk3-mediated necroptosis
Source: Cell Death Discov. 2022 Dec 2;8:477. doi: 10.1038/s41420-022-01272-2 (PMC9718801; doi:10.1038/s41420-022-01272-2)
Supplement: Supplementary file 3 — Suppl. Fig. 3 [file 41420_2022_1272_MOESM3_ESM.pdf]

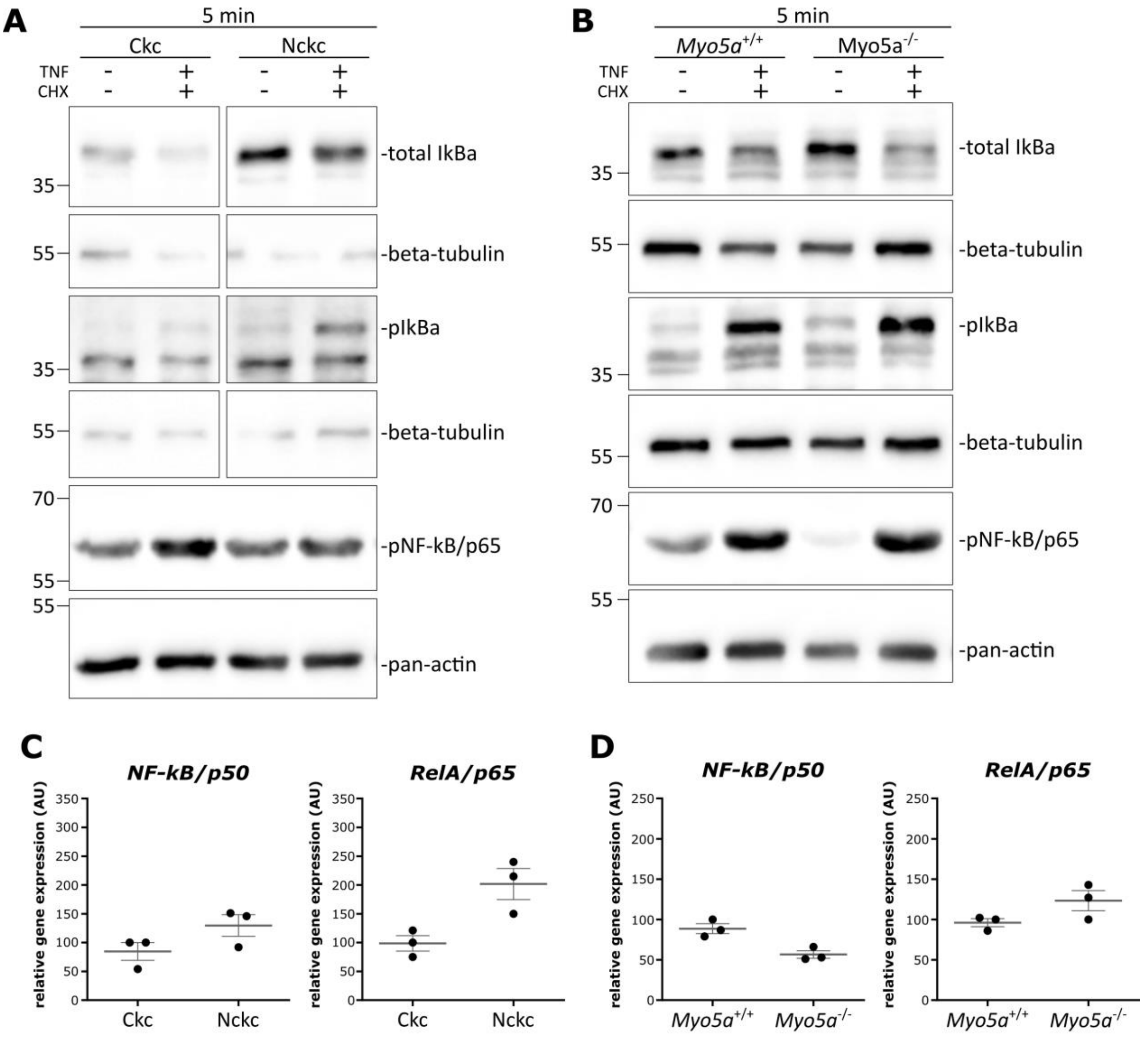


***Supplementary figure 3***

**Suppl. Fig. 3: Loss of cilia does not inhibit NF-kB signaling**
**(A,B)** Immunoblot analysis of **(A)** Ckc vs Nckc (n=3); **(B)** control vs *Myo5a*$^{-/-}$ , stimulated with TNFα and CHX for 5 min or either DMSO as control, stained for total IκBα (~40 kDa),pIκBα (~40 kDa) and pNF-κB. As housekeeping control either pan-actin (~44 kDa) or beta-tubulin (~55 kDa) were used (n=3). **(C,D)** Quantitative real-time PCR of NF-kB/p50 and RelA/p65 in mIMCD3 cells: **(C)** Ckc vs Nckc (n=3); **(D)** *Myo5a*$^{+/+}$ vs *Myo5a*$^{-/-}$ (n=3). Cells were treated with DMSO for 16 h. Statistical analysis was performed by using a one-way ANOVA followed by a two-sided Student's t-test (p-Value: >0.001***; 0.002**; 0.033*; ns=0.12).
