# Supplementary material for: Primary cilia suppress Ripk3-mediated necroptosis
Source: Cell Death Discov. 2022 Dec 2;8:477. doi: 10.1038/s41420-022-01272-2 (PMC9718801; doi:10.1038/s41420-022-01272-2)
Supplement: Supplementary file 4 — Suppl. Fig. 4 [file 41420_2022_1272_MOESM4_ESM.pdf]

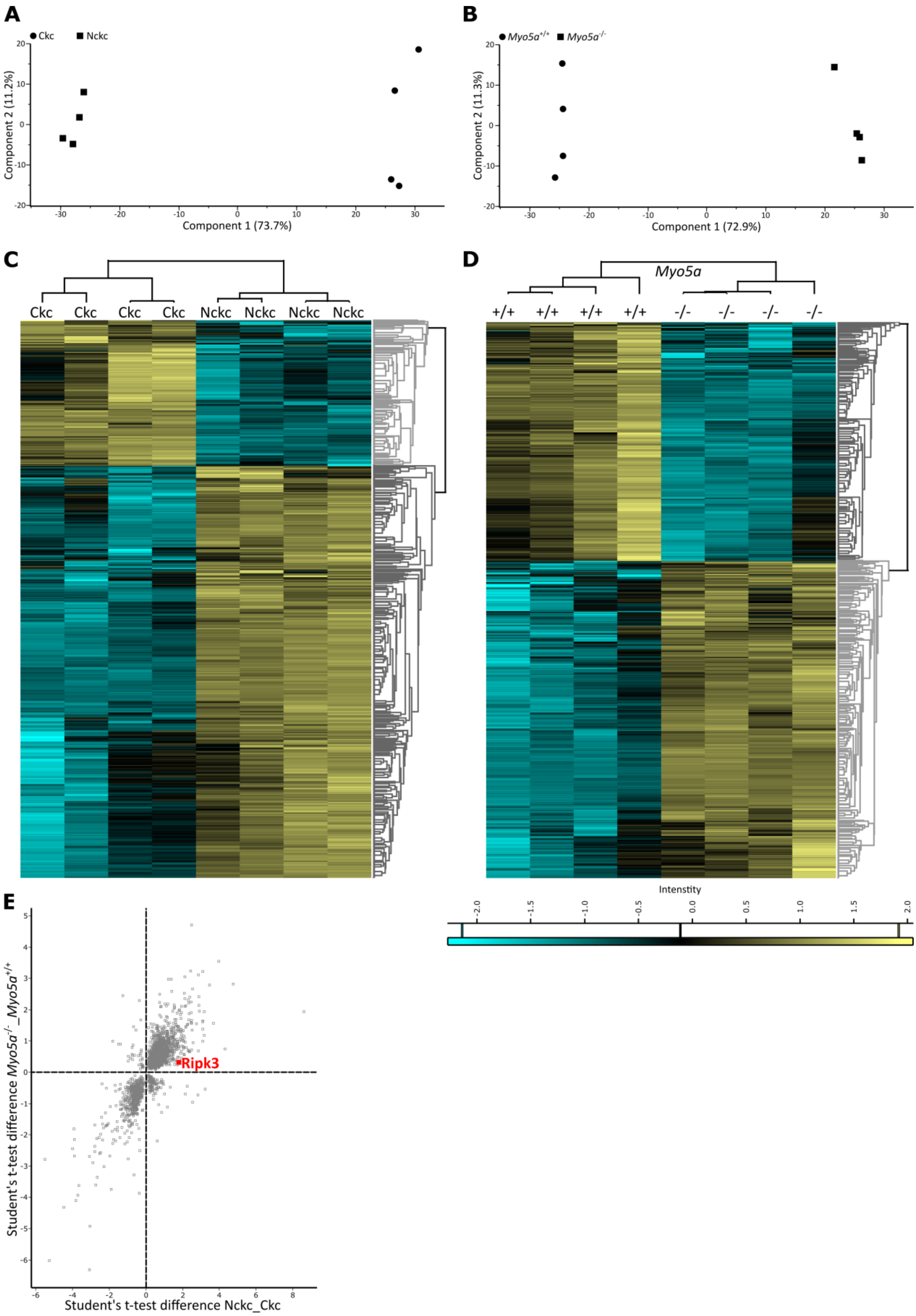


***Supplementary figure 4***

**Suppl. Fig. 4: Proteomic analyses: separation of ciliated from non-ciliated cells**
**(A, B)** Principal component analysis (PCA) plots of the protein expression data of ciliated vs. unciliated mIMCD3 cells. Depicted are the first two principal components. The axes represent the percentages of variation explained by the principal components. **(C,D)** Clustered heatmap based on log(2) LFQ values of identified differentially expressed proteins of Nckc versus Ckc (C) and *Myo5a*$^{-/-}$ versus *Myo5a*$^{+/+}$ cells (D), visualizing the differences among the groups of significantly upregulated (yellow) or downregulated (cyan) proteins. **(E)** Representative scatter plot of the combined data set visualizing only the significant regulated proteins for non-ciliated cells compared to ciliated cells. Depicted are the t-test differences of Nckc versus Ckc in protein expression on the x-axis and the t-test differences of *Myo5a*$^{-/-}$ versus *Myo5a*$^{+/+}$ control in protein expression on the y-axis.
