# Supplementary material for: Primary cilia suppress Ripk3-mediated necroptosis
Source: Cell Death Discov. 2022 Dec 2;8:477. doi: 10.1038/s41420-022-01272-2 (PMC9718801; doi:10.1038/s41420-022-01272-2)
Supplement: Supplementary file 5 — Suppl. Fig. 5 [file 41420_2022_1272_MOESM5_ESM.pdf]

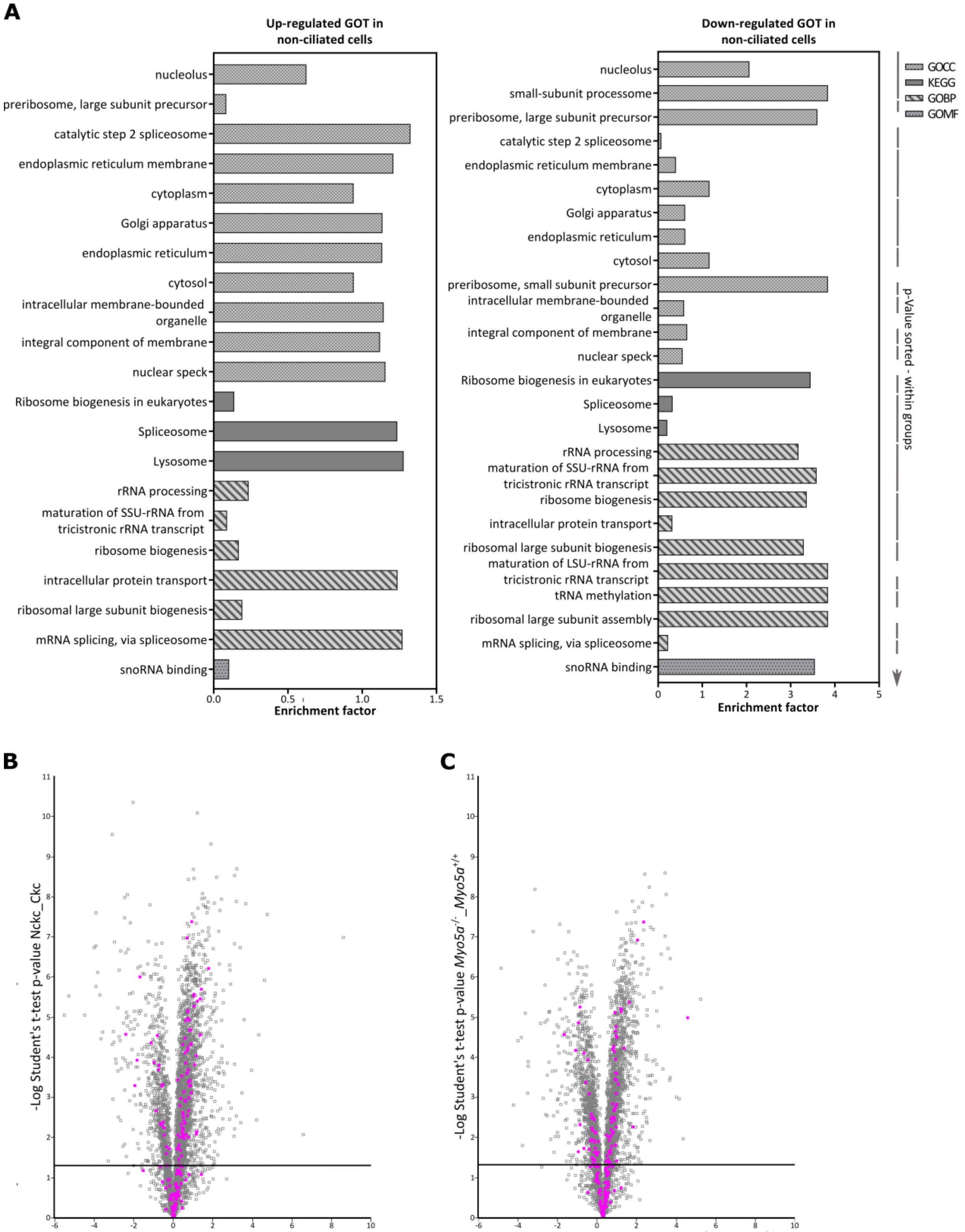


## *Supplementary figure 5*

**Suppl. Fig. 5: Shared proteomic alterations of non-ciliated cells**

**(A)** GO and KEGG pathway enrichment based on a Fisher exact test of the proteins found to be regulated in the combined non-ciliated data set, separated for up-and down-regulation. Ordered by p-values within the groups. **(B, C)** Representative volcano plots for Nckc (B) and *Myo5a*$^{-/-}$ (C), with the t-test differences in protein expression of both non-ciliated cell lines and their respective controls, on

the x-axis and the statistical significance (-log10 Student's t-test p-value) on the y-axis. Proteins associated with autophagy are highlighted in magenta.
