# Supplementary material for: Primary cilia suppress Ripk3-mediated necroptosis
Source: Cell Death Discov. 2022 Dec 2;8:477. doi: 10.1038/s41420-022-01272-2 (PMC9718801; doi:10.1038/s41420-022-01272-2)
Supplement: Supplementary file 6 — Suppl. Fig. 6 [file 41420_2022_1272_MOESM6_ESM.pdf]

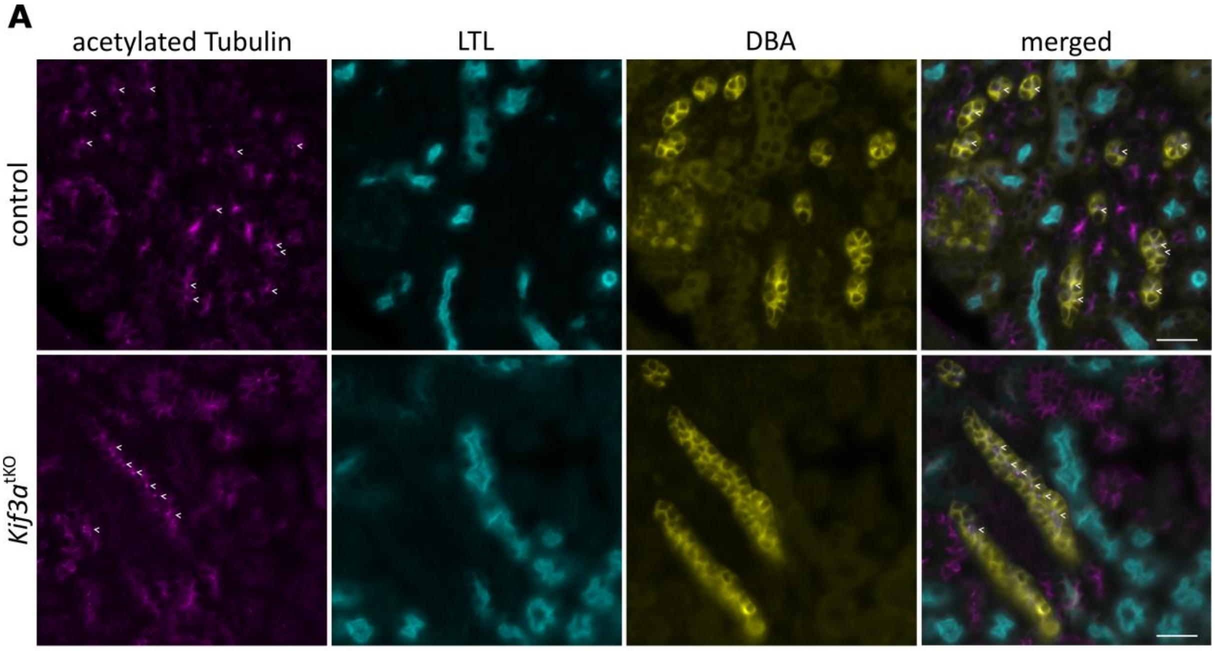

Supplementary figure 6

Suppl. Fig. 6: Primary cilia in distal tubules of Kif3a^{tko} at postnatal day P4
(A) Staining of primary cilia on paraffin-embedded kidney tissue revealed the presence of primary cilia in distal tubule in *Kif3a*$^{fl/fl}$:Ksp:cre$^{+/-}$ at postnatal day P4. Acetylated tubulin (magenta), LTL (proximal tubule marker; cyan), DBA (distal tubule marker; yellow) and nuclei (blue; scale bar 50 µm).
