# Supplementary material for: Primary cilia suppress Ripk3-mediated necroptosis
Source: Cell Death Discov. 2022 Dec 2;8:477. doi: 10.1038/s41420-022-01272-2 (PMC9718801; doi:10.1038/s41420-022-01272-2)
Supplement: Supplementary file 7 — Suppl. Fig. 7: original data file [file 41420_2022_1272_MOESM7_ESM.pdf]

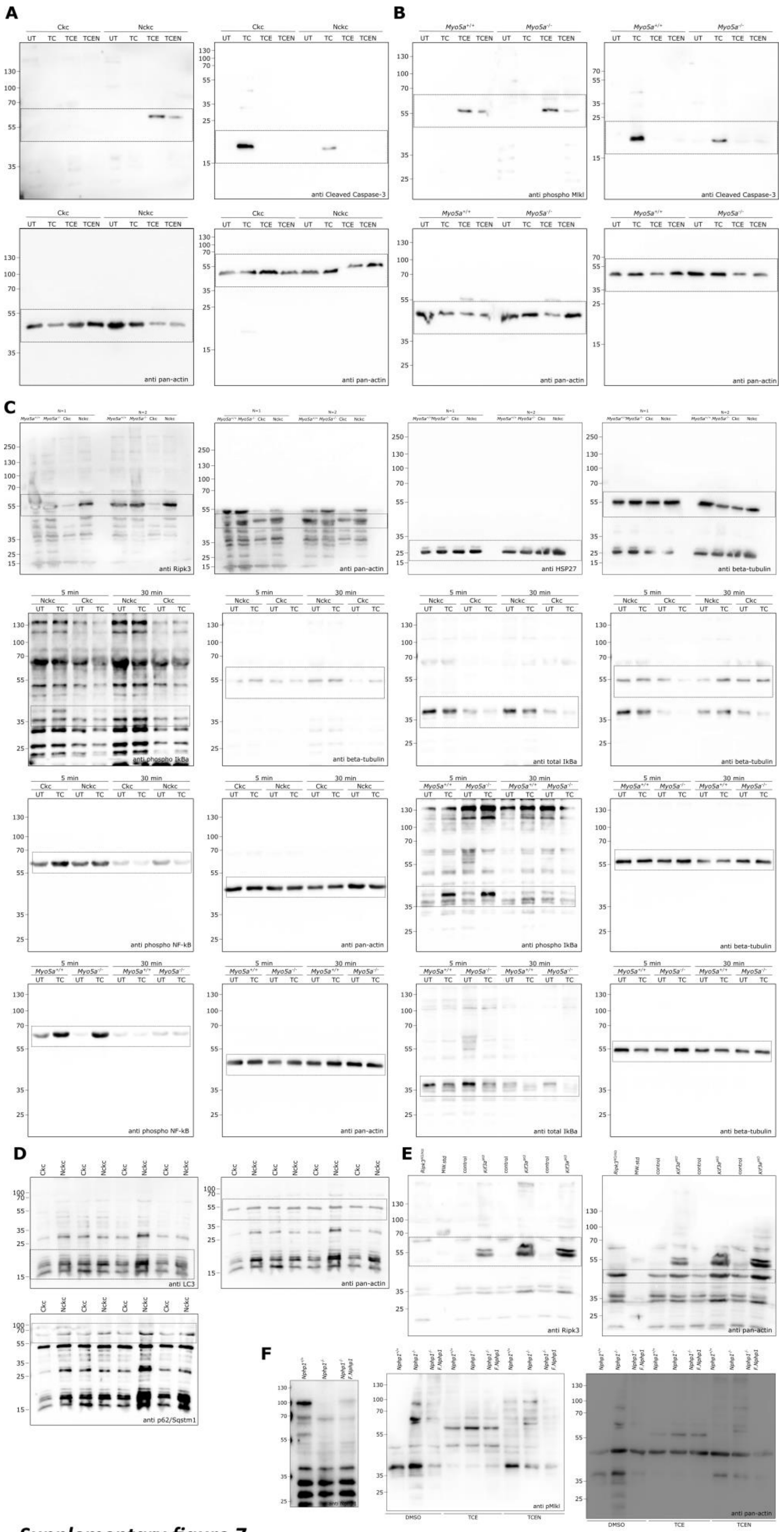

Supplementary figure 7

Suppl. Fig. 7: Original data: full-sized immunoblots

Original western blots, only cropped to gel size **(A)** of Figure 1, **(B)** of Figure 2, **(C)** of Figure 3, and Supplementary Figure 2 (one blot was covered by foil due to exposure issues), **(D)** of Figure 4 **(E)** of Figure 5 and **(F)** of Figure 6.
